# The P300 event-related potential as a physiological signature of cognitive reserve: links to working memory in aging

**DOI:** 10.3389/fpsyg.2026.1795165

**Published:** 2026-06-24

**Authors:** Agnese Ušacka, Marija Skrule, Kristīne Šneidere, Zigmunds Freibergs, Aivars Kaupužs, Ainārs Stepens

**Affiliations:** 1 Department of Health Psychology and Paedagogy, Riga Stradiņš University, Riga, Latvia; 2 Institute of Public Health, Riga Stradiņš University, Riga, Latvia; 3 Institute of Psychology, University of Tartu, Tartu, Estonia; 4 Riga Technical University Rezekne Academy, Rēzekne, Latvia; 5 Department of Education and Science, Riga East University Hospital, Riga, Latvia

**Keywords:** aging, cognitive reserve, electrophysiology, mild cognitive impairment, neural compensation, P300 ERP, working memory

## Abstract

Cognitive reserve (CR) may help explain why older adults differ in cognitive functioning despite comparable age- or pathology-related changes. However, the electrophysiological expression of CR across stages of cognitive decline remains unclear. This study examined whether CR predicts P300 event-related potential, and whether working memory (WM) capacity modulated the CR-P300 link across cognitive status groups. Seventy-one older adults [healthy controls (HC), *n* = 31, *M_age_* = 67.3, *SD_age_* = 7.19; mild cognitive impairment (MCI), *n* = 28, *M_age_* = 70.8, *SD_age_* = 7.06; moderate cognitive impairment (MoCI), *n* = 12, *M_age_* = 70.6, *SD_age_* = 6.50] completed a visuospatial WM task (Corsi block-tapping test). Using 32-channel EEG, P300 ERP responses were recorded during an auditory oddball task. CR was quantified using the Cognitive Reserve Index questionnaire, including education, working activity, and leisure activity domains, and global cognition was assessed using the Montreal Cognitive Assessment. Group-stratified hierarchical regression models tested (1) CR prediction of P300 amplitude and latency after adjusting for age and global cognition, and (2) CR × WM interaction. A whole-sample hierarchical regression model was used to test whether the CR-P300 amplitude association differed across cognitive status groups. In HC, CR positively predicted larger P300 amplitude. In MCI, the CR coefficient at mean WM was negative, whereas CR × WM interaction was positive, suggesting that CR-related amplitude patterns may depend on preserved WM capacity. The whole-sample model provided evidence that the CR-P300 amplitude association differed between HC and MCI. In MoCI, amplitude estimates were unstable and showed wide confidence intervals, supporting only exploratory interpretation. CR and CR × WM showed no reliable associations with P300 latency in HC or MCI. However, in MoCI, domain-level latency analyses, occupational and educational reserve showed comparatively larger negative coefficients, suggesting possible associations with shorter P300 latency. However, confidence intervals crossed zero. Thus, latency findings were treated as exploratory and less stable than amplitude effects. These findings suggest that the electrophysiological expression of CR may differ across cognitive status, with P300 amplitude showing preliminary evidence of CR-related variation in healthy aging and WM-dependent modulation in MCI. P300 latency appeared less sensitive to CR-related variation in the present sample.

## 1 Introduction

Aging is accompanied by progressive but highly heterogeneous changes in cognitive functioning. While many older adults experience declines in processing speed, working memory efficiency, and attentional control, others maintain relatively preserved cognitive performance well into late life, even in the presence of comparable age-related brain changes (Harada et al., 2013; Toepper, 2017). This inter-individual variability has motivated sustained interest in the concept of cognitive reserve (CR), which seeks to explain why similar levels of neural burden result in divergent cognitive outcomes (Stern, 2002; Stern et al., 2023).

Cognitive reserve refers to the brain’s capacity to optimize performance through efficient or flexible recruitment of cognitive strategies and neural networks, allowing individuals to cope more effectively with age-related changes and neuropathology (Stern et al., 2020). Originally introduced to explain discrepancies between observed brain pathology and clinical symptoms, CR is now understood as a multidimensional and dynamic construct shaped by lifelong experiences such as education, occupational complexity, and cognitively engaging leisure activities (Nucci et al., 2012; Stern, 2009). Higher CR has been associated with better cognitive functioning in aging and delayed clinical expression of neurodegenerative disease (Livingston et al., 2024; Stern et al., 2020). However, accumulating evidence indicates that CR does not confer uniform protection across all stages of cognitive decline. While higher CR may delay the clinical manifestation of cognitive impairment (e.g., Zhang et al., 2021), contemporary resilience models emphasize that such reserve reflects a finite and saturable compensatory capacity, which can be maintained only up to a threshold beyond which neuropathological burden can no longer be effectively offset (Masurkar et al., 2024). Once these compensatory mechanisms are exhausted, cognitive deterioration may proceed more rapidly – a phenomenon described as the “cognitive reserve paradox” (Scarmeas et al., 2001, 2006; Roe et al., 2010; van Loenhoud et al., 2019; Guo et al., 2025). These findings highlight CR as a dynamic process whose neural expression may differ across levels of aging and neurocognitive impairment.

Recent theoretical frameworks distinguish CR from related constructs such as brain reserve and brain maintenance, emphasizing that CR reflects functional adaptability rather than static structural capacity (Stern et al., 2019, 2020, 2023). This distinction underscores the need for methodological approaches capable of capturing functional neural processes that may be relevant to reserve and compensation. Behavioral measures alone provide limited insight into these latent mechanisms, particularly when cognitive performance remains within normative ranges despite underlying neural change (Cabeza et al., 2018; Stern et al., 2020). Functional neuroimaging studies have shown that individuals with higher CR may exhibit different patterns of task-related neural recruitment, including reduced activation that has been interpreted as greater neural efficiency, reflecting efficiency, or increased recruitment of additional networks that has been interpreted as compensatory processing (Cabeza et al., 2018; Anthony and Lin, 2017). However, such approaches are limited in their ability to resolve the temporal dynamics of these processes.

Electrophysiological measures, and event-related potentials (ERPs) in particular, offer a complementary avenue for investigating CR mechanisms with high temporal precision. Among ERP components, the P300 has been extensively studied in the context of aging and cognitive decline (Polich and Corey-Bloom, 2005; Polich et al., 1990; Costanzo et al., 2024; Pedroso et al., 2012). Elicited typically during oddball paradigms, the P300 is sensitive to attentional resource allocation, stimulus evaluation, and working memory updating (Polich, 2007). Its amplitude is commonly interpreted as an index of the amount of attentional and cognitive resources allocated to task-relevant stimuli, whereas its latency reflects the speed of stimulus evaluation and information processing (Polich, 2007; Verleger, 1997; Demirayak et al., 2023; Gu et al., 2017, 2018; Mendes et al., 2022). In cognitive aging research, larger P300 amplitude in the context of preserved or comparable behavioral task performance may be consistent with more effective engagement of task-related neural resources (Polich, 2007; Kok, 2001). However, P300 amplitude is not specific to neural efficiency and may also vary with attentional engagement, cognitive effort, and task demand (Donchin and Coles, 1988; Kok, 2001; Polich, 2007). In the context of cognitive reserve, P300 may therefore provide a temporally precise marker of task-related resource allocation that can be interpreted in relation to neural efficiency or compensatory engagement, particularly when considered alongside behavioral performance. Healthy aging is generally associated with reduced P300 amplitude and prolonged latency, reflecting altered attentional resource allocation and slower stimulus evaluation timing (van Dinteren et al., 2014). Importantly, variability in P300 characteristics has been linked to cognitive performance, risk of cognitive decline, and progression from mild cognitive impairment (MCI) to dementia (Olichney et al., 2022; Paitel et al., 2021; Costanzo et al., 2024).

Despite its conceptual alignment with CR, empirical findings on the relationship between CR and P300 characteristics remain inconsistent. Many studies rely on single proxies of CR, most often education, and do not account for the multidimensional nature of reserve (Šneidere et al., 2020; Hedges et al., 2016; Berezuk et al., 2021). Others employ tasks that heavily tax working memory to elicit the P300, making it difficult to disentangle whether observed associations reflect CR itself or task-specific cognitive demands (Khachatryan et al., 2021; Speer and Soldan, 2015). Moreover, P300 amplitude and latency are frequently treated interchangeably, despite theoretical reasons to expect that they index distinct aspects of neural and cognitive processes and may relate differently to CR across levels of cognitive decline (Khachatryan et al., 2021; Šneidere et al., 2020).

Working memory represents a particularly relevant cognitive domain in this context. As a core component of executive functioning, working memory is essential for goal-directed behavior and everyday cognition, and it is among the earliest functions to decline with aging (Toepper, 2017). Higher CR has been associated with better preserved working memory performance in both healthy aging and early cognitive decline (Lojo-Seoane et al., 2018; Mičič et al., 2020; Facal et al., 2014), suggesting that working memory may represent an important cognitive resource through which reserve-related processes are expressed. However, such reserve-related effects may not be uniform across levels of cognitive impairment. In individuals with greater cognitive impairment, the expression of CR-related neural differences may increasingly depend on the availability of preserved working memory resources and may become attenuated when these resources are substantially compromised (Cabeza et al., 2018; van Loenhoud et al., 2019).

Taken together, these considerations point to the need for integrative studies that examine cognitive reserve using complementary behavioral and neurophysiological measures, while explicitly accounting for cognitive capacity and cognitive status. Few studies have jointly examined multidimensional CR indices, electrophysiological markers, and working memory performance within the same individuals, and even fewer have addressed whether CR-related neural correlates differ across levels of cognitive impairment (Facal et al., 2014; Mičič et al., 2020; Khachatryan et al., 2021; Stern et al., 2020). Furthermore, it remains unclear whether P300 amplitude and latency, as distinct electrophysiological markers of attentional resource allocation and stimulus evaluation timing, relate differentially to CR across aging and neurocognitive impairment.

The present study addresses these gaps by investigating the associations between cognitive reserve, P300 electrophysiological markers, and working memory performance in older adults across cognitive status groups. Cognitive reserve was operationalized using a multidimensional index incorporating education, occupational activity, and leisure engagement (Nucci et al., 2012). P300 amplitude and latency were recorded during an oddball task and were treated as task-specific electrophysiological markers of attention-related resource allocation and stimulus evaluation timing, respectively. Working memory capacity was assessed using a visuospatial span measure. Based on contemporary cognitive reserve frameworks, we hypothesized that higher cognitive reserve would be associated with larger P300 amplitude in healthy older adults, a pattern that may be consistent with more effective engagement of task-related neural resources when behavioral performance is comparable. In individuals with mild cognitive impairment, we expected CR-P300 amplitude associations to vary as a function of working memory capacity, consistent with possible working memory dependent expression of reserve-related processing. Given theoretical links between cognitive reserve, processing speed, and compensatory slowing, we further examined whether P300 latency would relate to CR across cognitive status groups. Finally, in individuals with moderate cognitive impairment, we expected CR-related electrophysiological associations to be weaker, more variable or no longer detectable. Given the cross-sectional design, these hypotheses were framed as cognitive-status-related differences.

By integrating multidimensional CR indices, working memory performance and P300 electrophysiological measures, the present study aims to clarify whether CR-related neural correlates differ across cognitive status groups. Rather than treating P300 amplitude and latency as interchangeable markers, the study examines whether these ERP features provide distinct information about attention-related resource allocation and stimulus evaluation timing. In doing so, this work contributes to a more nuanced understanding of how cognitive reserve may be expressed in task-related neural processing during aging and neurocognitive impairment.

## 2 Materials and methods

### 2.1 Participants and procedure

Seventy-one older adults participated in the study (63% women). Participants were recruited through community sampling, non-governmental organizations, community outreach initiatives, and clinical referrals. The sample included participants referred from clinical practice as well as community-dwelling older adults who volunteered to participate in the study.

Participants were classified into three cognitive status groups: healthy controls (HC; *n* = 31, *M* = 67.3, *SD* = 7.19), mild cognitive impairment (MCI; *n* = 28, *M* = 70.8, *SD* = 7.06), and moderate cognitive impairment (MoCI; *n* = 12, *M* = 70.6, *SD* = 6.50). Inclusion criteria comprised older age (≥55), capacity to provide informed consent, and sufficient sensory abilities to complete neuropsychological testing and EEG procedures. Because the P300 was elicited using an auditory oddball paradigm, participants were required to have hearing sufficient to perceive the auditory stimuli and follow task instructions. Exclusion criteria included major neurological disorders (e.g., stroke, epilepsy, and traumatic brain injury), severe psychiatric conditions, significant uncorrected sensory impairments, including hearing difficulties that would interfere with auditory task performance, or other medical conditions known to substantially affect cognitive functioning.

Cognitive status classification was determined by a neurologist and a clinical psychologist based on expert clinical consensus informed by standardized neuropsychological testing, neurological examination, and neuroimaging findings, including magnetic resonance imaging (MRI). The neuropsychological assessment included the Montreal Cognitive Assessment (MoCA, Nasreddine et al., 2005), although group assignment was not based on MoCA score alone. MCI was defined according to International Classification of Diseases, 10th Revision (ICD-10) criteria as cognitive impairment evident in at least one cognitive domain (World Health Organization, 2019). The MoCI group included individuals showing more substantial cognitive deficits, with impairment evident in at least three cognitive domains on clinical evaluation. Individuals with clinically diagnosed dementia were not included in the analytic sample because reliable completion of the interview-based cognitive reserve assessment could not be ensured. Group assignment therefore reflects clinically informed cognitive status rather than screening scores alone.

Data collection followed a broadly standardized order. Participants first completed the clinical and neuropsychological assessment, including measures of global cognitive functioning and working memory. The EEG session was conducted after the cognitive assessment and included the auditory oddball task used to elicit the P300 component. The cognitive reserve assessment was administered after the main cognitive and EEG assessments, either in person as the final assessment of the study visit or, when participants reported fatigue or preferred to complete it later, during a separate structured telephone interview. In both formats, the assessment followed the same standardized administration and scoring procedures. All participants provided written informed consent prior to participation. The study protocol was approved by the Central Medical Ethics Committee of the Republic of Latvia.

### 2.2 Psychological and cognitive assessments

Global cognitive functioning was assessed using the MoCA (version 8.1, Nasreddine et al., 2005), a widely used screening instrument designed to detect mild cognitive impairment and early cognitive decline. The MoCA was administered individually following standardized administration procedures. Although MoCA scores were not used as the sole criterion for cognitive status classification, they were included as a continuous covariate in all statistical analyses to account for interindividual differences in overall cognitive performance.

Cognitive reserve was assessed using the Cognitive Reserve Index questionnaire (CRIq; Nucci et al., 2012). The CRIq is a structured interview-based instrument that quantifies lifetime cognitive engagement across three domains: education, occupational attainment, and leisure activities. Information on formal education, work history, and cognitively stimulating leisure activities was collected either in person or via telephone interview following cognitive testing and EEG recording session. The Education subscale captures formal educational attainment and training duration, the Working Activity subscale indexes occupational complexity and duration across adulthood, and the Leisure Time subscale assesses engagement in cognitively, socially, and intellectually stimulating activities during adulthood and later life. Subscale scores were calculated according to standardized CRIq scoring procedures and combined into a total CR index score. Higher CRIq scores indicate greater accumulated cognitive reserve.

Working memory capacity was assessed using the visuospatial Corsi block-tapping task (Corsi, 1972), a well-established measure of visuospatial working memory. During the task, participants were presented with sequences of spatially distributed blocks that were highlighted one at a time and were instructed to reproduce each sequence in the same order immediately after presentation. Sequence length increased progressively until the participant was no longer able to correctly reproduce the sequence.

Working memory performance was indexed using the maximum correctly reproduced sequence length (Corsi span), which reflects the capacity to temporarily maintain and manipulate visuospatial information. The Corsi task was selected because it provides a modality-independent measure of working memory capacity that is not directly confounded with the auditory oddball task used to elicit the P300, thereby allowing a clearer examination of working memory as a moderating cognitive resource rather than as a task-specific demand.

### 2.3 EEG task and data acquisition

The EEG was recorded using a g.tech g. Nautilus 32-channel wireless EEG system with electrodes positioned according to the international 10–20 system and with the left earlobe electrode as reference. Signals were digitized at a sampling rate of 500 Hz. Electrode impedances were kept below 50 kΩ, in accordance with manufacturer specifications for the active electrode EEG system used. Higher impedance thresholds are acceptable for active electrode systems because the preamplification at the electrode reduces susceptibility to noise relative to passive systems. EEG data were acquired using g. Recorder software while participants were seated comfortably in a quiet, dimly lit room.

The EEG data were recorded during an auditory oddball task designed to elicit the P300 (P3b) component. Two types of auditory stimuli were presented: a frequent standard tone (1,000 Hz; 80% probability) and an infrequent target tone (2000 Hz; 20% probability). Stimuli were 100 ms in duration, presented at approximately 100 dB, with interstimulus intervals jittered between 1.5 and 2.0 s. Before EEG recording, participants were asked to confirm that the auditory stimuli were clearly audible and that they could distinguish and respond to the target tones. Stimulus presentation order was randomized. A total of 300 tones were presented, yielding approximately 60 target trials per participant and resulting in a task duration of approximately 10.5 min. Participants were instructed to respond to target stimuli by pressing a response key with their dominant hand while ignoring standard tones. Behavioral responses were monitored to ensure task engagement.

### 2.4 EEG preprocessing and ERP extraction

The EEG preprocessing was performed in MATLAB (R2022b; The MathWorks, Inc., 2022) using the EEGLAB toolbox (Delorme and Makeig, 2004). EEG signals were band-pass filtered between 0.3 and 40 Hz and were re-referenced offline to the average reference. Channels affected by excessive artifacts were interpolated. Ocular and muscle artifacts were identified and removed using independent component analysis (ICA). The continuous EEG signal was segmented into epochs from −300 ms to 800 ms relative to target stimulus onset and baseline-corrected using the pre-stimulus interval. For each participant, averaged ERPs were computed from artifact-free epochs.

The P300 (P3b) component was quantified primarily at the Pz electrode, consistent with established P300 literature and with visual inspection of individual and grand-average waveforms indicating a clear parietal maximum. Pz was therefore retained as the primary electrode for hypothesis-driven analyses in order to maximize component reliability and limit the number of primary statistical tests. To characterize the spatial distribution of the component and evaluate whether the observed effects extended to adjacent parietal sites, P300 amplitude and latency were also extracted at P3 and P4. These adjacent-electrode analyses were treated as secondary spatial sensitivity analyses. Grand-average scalp topographical maps were generated for the P300 time window to descriptively verify the spatial distribution of the component. These maps were used to support interpretation of the electrode-specific analyses rather than as separate inferential tests. P300 amplitude was defined as the maximum positive voltage within the 250–650 ms post-stimulus window. P300 latency was defined as the time point of this maximum positivity. This time window was selected to accommodate age-related variability in P300 latency and to ensure reliable component identification across cognitive status groups.

### 2.5 Data analytic strategy

All statistical analyses were conducted in RStudio (version 2024.12.1 + 563; Posit Team, 2024) using the tidyverse (Wickham et al., 2019) and boot (Canty and Ripley, 2023) packages. The primary analyses examined whether CR was associated with P300 ERP amplitude and latency at Pz electrode within each cognitive status group, and whether this association varied as a function of WM capacity. Accordingly, hierarchical linear regression models were estimated separately within each cognitive status group: healthy controls (HC), mild cognitive impairment (MCI), and moderate cognitive impairment (MoCI).

Prior to model estimation, all continuous predictors including age, MoCA total score, CRIq total score, and working memory performance derived from the Corsi block-tapping task were standardized as *z*-scores. Standardization was applied to facilitate interpretability of regression coefficients, ensure comparability across predictors, and reduce potential multicollinearity when testing interaction effects. The CR × WM interaction term was computed by multiplying standardized CRIq total scores by standardized Corsi scores.

Model estimation followed a hierarchical entry procedure. In Step 1, age and MoCA scores were entered as covariates to account for chronological aging and global cognitive functioning. In Step 2, CRIq total score was added to assess the main effect of cognitive reserve on ERP outcomes beyond age and overall cognition. In Step 3, the CR × WM interaction term was introduced to test whether the association between CR and P300 outcomes varied as a function of WM capacity. Separate models were estimated for two dependent variables – P300 amplitude, treated as the primary electrophysiological outcome, and P300 latency, treated as a secondary electrophysiological outcome. To examine whether the primary Pz amplitude findings were spatially constrained or also observable at adjacent parietal electrodes, sensitivity analyses were conducted for P300 amplitude at P3 and P4.

To evaluate whether CR-WM-P300 associations differed across cognitive status groups, we additionally estimated a whole-sample hierarchical regression model including cognitive status group, CRIq total score, WM capacity, and their interaction terms, including the Group × CR × WM interaction at Pz electrode. Age and MoCA were retained as covariates. The whole-sample model was used as a between-group comparison of regression slopes and moderation effects.

Given the modest sample sizes within groups, particularly in the MoCI group, and the potential for violations of classical linear model assumptions, regression coefficients and confidence intervals were estimated using non-parametric bootstrapping with 5,000 resamples. Effects were interpreted as statistically supported when bootstrapped 95% confidence intervals did not include zero. Parametric *p*-values were reported where appropriate but interpreted alongside bootstrapped confidence intervals. To evaluate multiplicity across theoretically relevant regression coefficients, Benjamini–Hochberg false discovery rate (FDR) correction was applied within analysis families. Model fit and exploratory contribution were summarized using *R*^2^, Δ*R*^2^, and unique *R*^2^ estimates. MoCI analyses were considered exploratory because of the sample size and were interpreted primarily in terms of estimate uncertainty rather than definitive evidence of effects.

## 3 Results

### 3.1 Sample characteristics and descriptive statistics

Demographic, cognitive reserve, working memory, behavioral oddball task performance and electrophysiological characteristics of the study sample by cognitive status group are summarized in Table 1. The three groups did not differ significantly in age or sex distribution. As expected, global cognitive functioning indexed by MoCA scores differed significantly across groups, with progressively lower scores from HC to MCI and MoCI. Groups also differed in selected cognitive reserve and working memory indices. Specifically, CRIq education scores differed across groups, with post-hoc Games-Howell comparisons indicating lower scores in MoCI compared with HC (*p* = 0.002) but not compared with MCI (*p* = 0.176). Working memory capacity (Corsi span) also differed across groups, with lower Corsi span in MoCI compared with both HC (*p* = 0.018) and MCI (*p* = 0.025). The total CRIq and the remaining CRIq subscales showed no significant group differences. Oddball task accuracy and reaction time did not differ significantly across groups, suggesting broadly comparable behavioral task performance during EEG recording. P300 amplitude and latency did not differ significantly across groups at Pz, P3, or P4.

**Table 1:** Demographic, cognitive reserve, working memory, and electrophysiological characteristics of the study sample by cognitive status group.

Variable	HC (*n* = 31)	MCI (*n* = 28)	MoCI (*n* = 12)	*p*-value
Age	67.3 (7.19)	70.8 (7.70)	70.6 (6.50)	0.154
Sex (m/f)	11/20	11/17	4/8	0.948
MoCA	26.10 (1.30)	22.57 (2.56)	17.33 (3.77)	<0.001
CRIq	130.16 (18.90)	128.96 (31.05)	111.17 (23.58)	0.08
CRIq_education_	125.52 (16.39)	119.00 (24.83)	108.58 (11.33)	0.009
CRIq_occupation_	126.39 (20.31)	127.25 (32.32)	114.17 (26.89)	0.269
CRIq_leisure_	118.16 (20.33)	121.32 (28.64)	103.25 (26.18)	0.197
WM capacity	3.65 (2.15)	3.50 (1.84)	1.58 (1.98)	0.011
Oddball task accuracy	299 (1.15)	299 (1.29)	297 (4.01)	0.341
Oddball task RT	400 (120)	440 (130)	460 (210)	0.400
P300 amplitude Pz	4.08 (2.51)	4.80 (2.63)	4.08 (3.57)	0.352
P300 latency Pz	461 (117)	486 (106)	431 (99)	0.291
P300 amplitude P3	3.64 (2.32)	4.50 (2.31)	3.48 (2.50)	0.302
P300 latency P3	457 (102)	451 (114)	460 (111)	0.961
P300 amplitude P4	3.86 (2.36)	4.64 (2.49)	2.65 (2.28)	0.066
P300 latency P4	460 (104)	489 (101)	489 (108)	0.517

Values are presented as mean and standard deviation in parentheses. HC = healthy controls; MCI = mild cognitive impairment; MoCI = moderate cognitive impairment; MoCA = Montreal Cognitive Assessment; CRIq = Cognitive Reserve Index questionnaire; WM = working memory (Corsi block-tapping span). Oddball task accuracy reflects the number of correct responses out of 300 trials, including correct responses to target tones and correctly withheld responses to non-target tones. Oddball task RT reflects mean reaction time in milliseconds to correctly detected target tones in milliseconds. P300 amplitude values measured as microvolts (μV) and P300 latency as milliseconds (ms). *p*-values reflect group comparisons (one-way ANOVA or Kruskal–Wallis tests, depending on distribution; *χ*^2^ test for sex).

### 3.2 Cognitive reserve, working memory, and P300 amplitude at Pz

**Figure 1:**
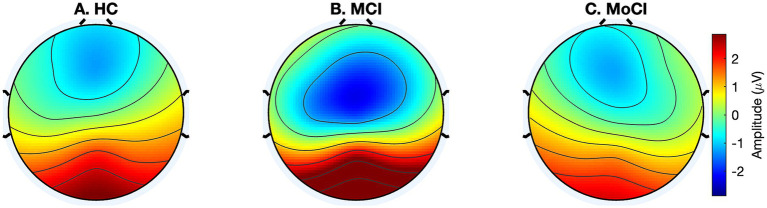
Grand-average scalp topographies of the target-evoked P300 response in healthy controls (**A**; HC), individuals with mild cognitive impairment (**B**; MCI), and individuals with moderate cognitive impairment (**C**; MoCI), averaged across the 250–600 ms time window. All panels are displayed using the same color scale. Warmer colors indicate more positive amplitudes.

Before testing cognitive reserve – P300 association, grand-average scalp topographies were inspected to characterize the spatial distribution of the target-evoked P300 response. Across cognitive status groups, the P300 showed a posterior parietal distribution, with the strongest positive activity observed over posterior scalp regions (Figure 1). This pattern supported the use of Pz as the primary electrode for hypothesis-driven analyses and motivated secondary analyses at adjacent parietal electrodes P3 and P4.

Hierarchical regression analyses were conducted separately within each cognitive status group to examine whether cognitive reserve predicted P300 amplitude, after controlling for age and global cognitive functioning, and whether this association was moderated by working memory capacity. Regression coefficients for predictors of theoretical interest are reported in Table 2.

**Table 2:** Group-stratified hierarchical regression models predicting P300 amplitude at Pz.

Group	Predictor	*B*	95% CI	*p*-value	*p*FDR	*R*^2^ full model	Unique *R*^2^
HC	CR total	1.35	[0.14, 2.65]	0.039	0.125	0.154	0.149
HC	CR × WM	−0.16	[−1.70, 0.87]	0.788	0.788	0.154	0.002
MCI	CR total	−0.94	[−2.06, −0.27]	0.037	0.125	0.225	0.166
MCI	CR × **WM**	1.26	[0.32, 3.11]	0.083	0.166	0.255	0.111
MoCI	CR total	−0.93	[−8.74, 4.68]	0.660	0.788	0.409	0.018
MoCI	CR × WM	0.49	[−4.44, 4.18]	0.712	0.788	0.409	0.012

*B* values represent unstandardized regression coefficients for P300 amplitude (μV) associated with a one standard deviation change in the predictor. Confidence intervals are bootstrapped (5,000 resamples). pFDR are Benjamini–Hochberg false discovery rate-adjusted *p*-values across the theoretically relevant coefficients in the primary group-stratified amplitude models. *R*^2^ full model refers to the final Step 3 model including age, MoCA, CR total, and the CR × WM interaction. For CR total, the final column reports unique *R*^2^ in the Step 3 model, for CR × WM it corresponds to the additional variance explained by adding the interaction term at Step 3.

#### 3.2.1 Healthy controls

**Figure 2:**
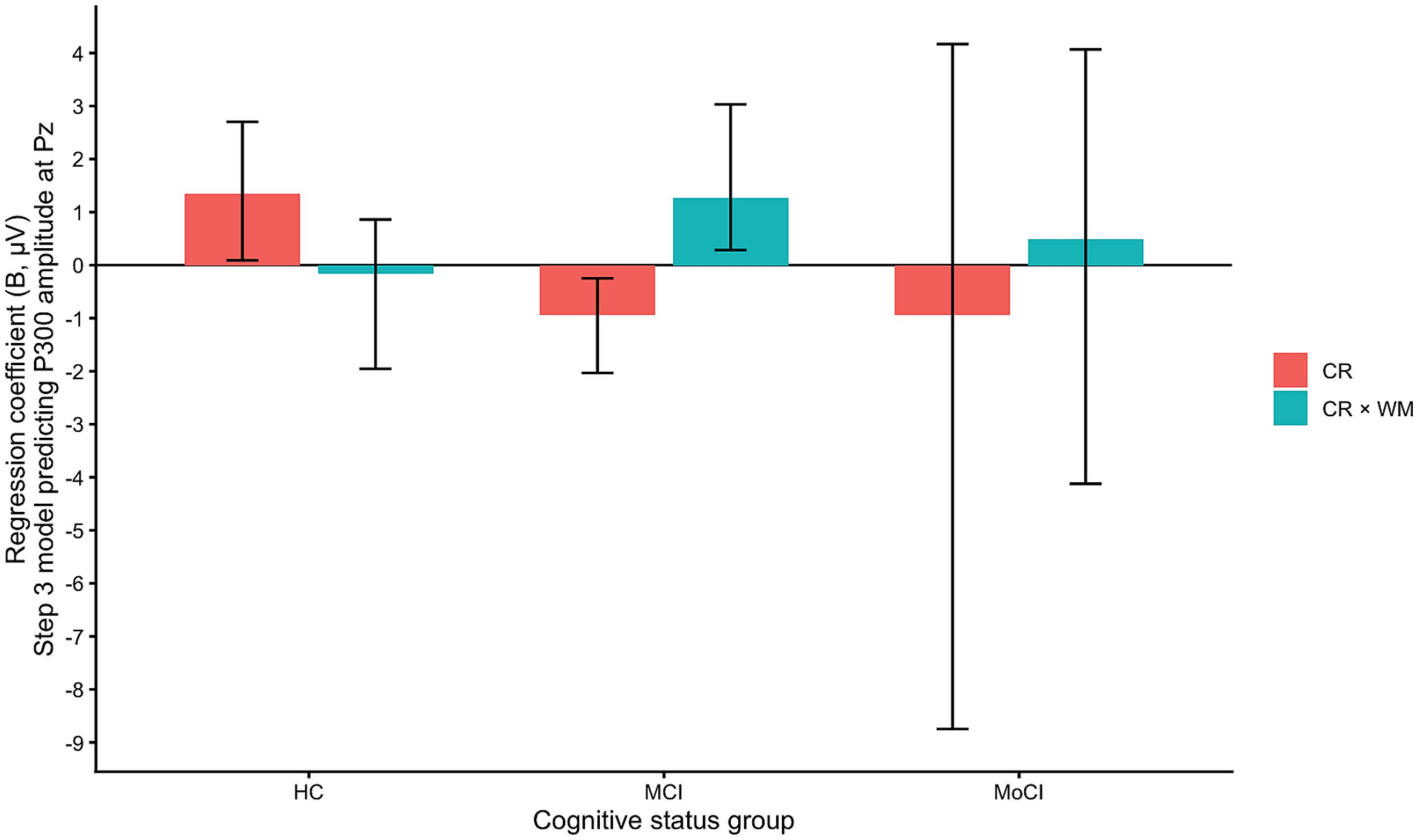
Group-stratified Step 3 regression coefficients for predictors of P300 amplitude at Pz. Bars represent unstandardized regression coefficients (B), indicating change in P300 amplitude in μV associated with a one standard deviation increase in the predictor. Error bars indicate bootstrapped 95% confidence intervals based on 5,000 resamples. CR total represents the association between cognitive reserve and P300 amplitude at mean working memory. CR × WM represents the interaction between cognitive reserve and working memory.

In healthy older adults, age and MoCA scores entered at Step 1 explained little variance in P300 amplitude at Pz. However, adding CR total score at Step 2 increased explained variance by 14.7%, indicating a non-trivial contribution of cognitive reserve beyond age and global functioning. In the final Step 3 model, higher CR total score remained positively associated with P300 amplitude (*B* = 1.35, 95% CI [0.14, 2.65]), with bootstrapped 95% confidence intervals excluding zero and a unique variance contribution of 14.9%. However, this effect did not survive Benjamini-Hochberg FDR correction (*p*FDR = 0.125) and should therefore be interpreted cautiously. The CR × WM interaction was not supported (*B* = −0.16, 95% bootstrap CI [−1.70, 0.87]), as its confidence intervals crossed zero and the interaction explained negligible additional variance. Thus, in HC, the pattern suggested a positive CR-P300 amplitude association that did not depend on WM capacity (Figure 2).

#### 3.2.2 Mild cognitive impairment

**Figure 3:**
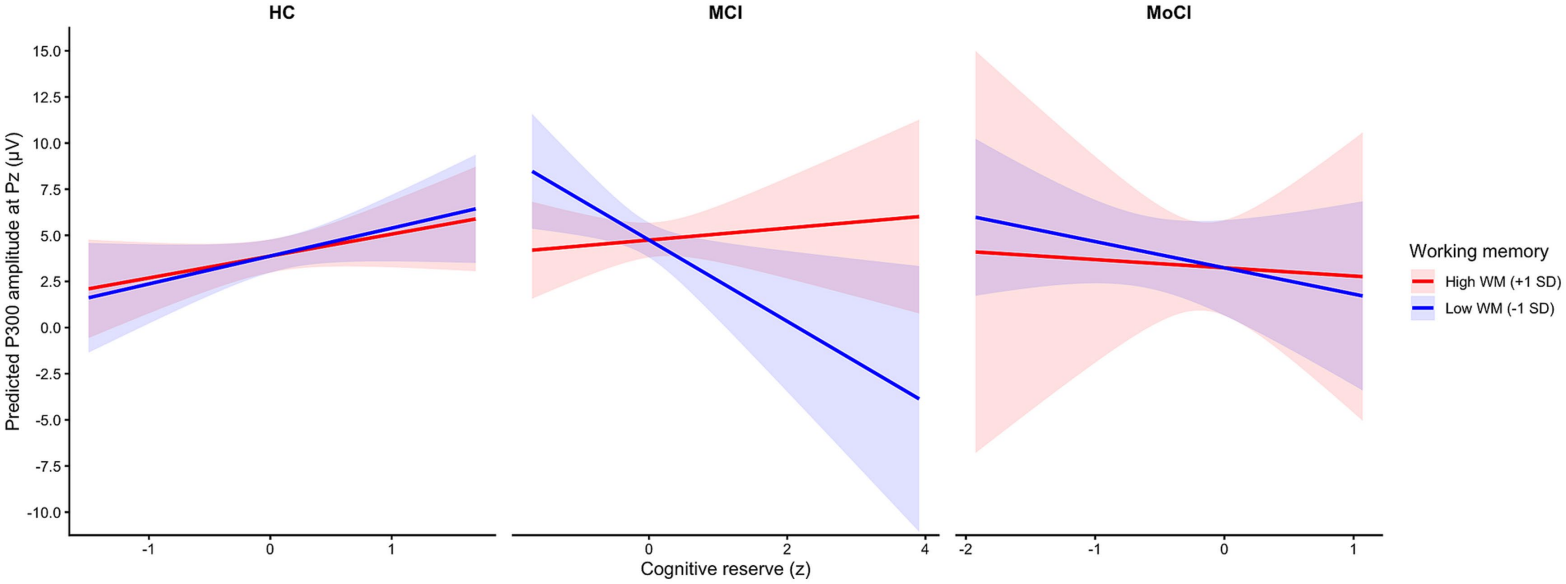
Model-predicted P300 amplitude at Pz as a function of cognitive reserve and working memory across cognitive status groups. Lines represent predicted values from group-specific Step 3 hierarchical regression models, with age and MoCA held constant. Predictors are shown separately for low (−1 SD) and high (+1 SD) working memory capacity and plotted across the observed cognitive reserve range within each group. Shaded areas indicate model-based 95% confidence intervals.

In the MCI group, cognitive reserve showed a negative association with P300 amplitude in the final Step 3 model (*B* = −0.94, 95% CI [−2.06, −0.27]), with a unique variance contribution of 16.6%. Importantly, the interaction between cognitive reserve and working memory was positive (*B* = 1.26), with a bootstrapped confidence interval that narrowly exceeded zero (95% CI [0.32, 3.11]), suggesting that the association between cognitive reserve and P300 amplitude varied as a function of working memory capacity. At the model level, adding CR × WM interaction explained additional 11.1% of variance in P300 amplitude, indicating a non-trivial conditional effect. However, the main effect of cognitive reserve and the interaction did not survive FDR correction and should therefore be interpreted cautiously. Model predicted simple slopes illustrated that the CR-P300 association was more positive among individuals with higher working memory capacity and more negative among those with lower working memory capacity (Figure 3). Thus, the MCI pattern was consistent with possible WM-dependent expression of cognitive reserve (Table 3).

**Table 3:** Whole-sample group comparison model predicting P300 amplitude at Pz.

Effect	*B*	95% CI	*p*-value	*p*FDR
CR in HC	1.41	[0.17, 2.62]	0.04	0.119
MCI vs. HC difference in CR	−2.36	[−4.05, −0.97]	0.005	0.031
MoCI vs. HC difference in CR	−1.25	[−7.11, 5.45]	0.439	0.659
CR × WM in HC	−0.19	[−1.74, 0.83]	0.761	0.761
MCI vs. HC difference in CR × WM	1.52	[0.19, 3.86]	0.123	0.246
MoCI vs. HC difference in CR × WM	0.50	[−3.99, 5.26]	0.662	0.761

HC – the reference group; “CR in HC” and “CR × WM in HC” represent the reference group effects; MCI and MoCI rows represent differences from HC; *B* values are unstandardized regression coefficients for P300 amplitude in μV; *p*-values are uncorrected coefficient-level values; *p*FDR values are Benjamini–Hochberg false discovery rate-adjusted values across six theoretically relevant whole-sample amplitude effects.

#### 3.2.3 Moderate cognitive impairment

In the MoCI group, neither cognitive reserve nor the CR × WM interaction showed a robust association with P300 amplitude at Pz. Confidence intervals for both predictors were wide and spanned zero (CR total: *B* = −0.93, 95% CI [−8.74, 4.68]; interaction: *B* = 0.49, 95% CI [−4.44, 4.18]). Variance-explained estimates also indicated negligible unique or incremental contributions, with CR total explaining 1.8% unique variance in the final model and the interaction adding only 1.2% additional variance. Thus, unlike the patterns observed in HC and MCI, the MoCI models did not provide reliable evidence for CR-related or WM-dependent modulation of P300 amplitude. Given the small MoCI sample and substantial estimate uncertainty, these findings should be interpreted as exploratory.

#### 3.2.4 Whole-sample group interaction model for P300 amplitude at Pz

To formally test whether the CR-P300 amplitude associations differed across cognitive status groups, a whole-sample hierarchical regression model was estimated with HC as the reference group. The model included age and MoCA as covariates, group, CR total, Group × CR terms, CR × WM, and Group × CR × WM terms. The final model explained 18.3% of the variance in P300 amplitude at Pz. Adding the Group × CR block significantly improved model fit (ΔR^2^ = 0.094, *p* = 0.039), and this block explained 11.7% unique variance in the final model. In contrast, adding the CR × WM and Group × CR × WM terms explained an additional 4.7% of variance but did not significantly improve model fit (*p* = 0.339).

At the coefficient level, the CR effect in HC was positive and its bootstrapped confidence interval excluded zero (*B* = 1.41, 95% CI [0.17, 2.62]), although this effect did not survive FDR correction (*p*FDR = 0.119). Importantly, the MCI versus HC difference in the CR effect was negative, with confidence interval excluding zero (*B* = −2.36, 95% CI [−4.05, −0.97]), and remained significant after the FDR correction (*p*FDR = 0.031). This indicates that the CR-P300 amplitude association differed reliably between HC and MCI. The corresponding MoCI versus HC contrast was not reliable (*B* = −1.25, 95% CI [−7.11, 5.45]). The MCI versus HC difference in the CR × WM effect also showed a confidence interval excluding zero (*B* = 1.52, 95% CI [0.19, 3.86]) but did not survive multiple-comparison correction (*p*FDR = 0.246). Therefore, the whole-sample model provided reliable corrected evidence for a group difference in the CR-P300 amplitude association between HC and MCI, whereas evidence for group differences in WM-dependent moderation was weaker and should be interpreted cautiously.

### 3.3 Adjacent-electrode analyses at P3 and P4

Because the grand-average topographies showed a posterior parietal P300 distribution across groups (Figure 1), secondary analyses were conducted at the adjacent parietal electrodes P3 and P4 to evaluate whether the primary Pz findings extended beyond the midline parietal site. These analyses were treated as spatial sensitivity analyses rather than additional primary tests.

Overall, these analyses did not provide consistent evidence that the CR-P300 or CR × WM effects generalized beyond Pz. At P3, none of the theoretically relevant effects were robust after bootstrapping or FDR correction. In HC, the CR effect was positive but uncertain, (*B* = 1.07, 95% CI [−0.07, 2.65]), and the CR × WM interaction was not supported (*B* = 0.30, 95% CI [−1.66, 1.34]). In MCI, both the CR effects (*B* = −0.10, 95% CI [−0.88, 1.25]) and CR × WM interaction (*B* = −0.01, 95% CI [−1.66, 1.64]), were close to zero. In MoCI, estimates were again unstable and confidence intervals were wide, with both CR (*B* = −1.83, 95% CI [−5.58, 6.56]), and CR × WM (*B* = −1.48, 95% CI [−3.91, 3.00]) crossing zero.

At P4, HC and MCI effects were similarly not robust. In HC, neither CR (*B* = 0.98, 95% CI [−0.19, 2.09]) nor CR × WM (*B* = 0.13, 95% CI [−0.89, 0.98]) showed reliable associations with P300 amplitude. In MCI, the CR effect was near zero (*B* = −0.13, 95% CI [−0.85, 1.35]) and the CR × WM interaction was positive but uncertain (*B* = 0.69, 95% CI [−0.89, 1.96]). In MoCI, the P4 coefficients for CR (*B* = 3.28, 95% CI [−1.48, 7.30]) and CR × WM (*B* = 1.78, 95% CI [−1.00, 4.45]) were larger in magnitude but had wide confidence intervals that crossed zero. Thus, the adjacent-electrode analyses did not reproduce the primary Pz pattern in a robust or spatially consistent manner. These findings support retaining Pz as the primary electrode for interpretation, while suggesting that the observed CR-related amplitude effects were most clearly expressed at the parietal midline site.

### 3.4 Cognitive reserve and P300 latency

Parallel group-stratified hierarchical regression models were conducted with P300 latency at Pz as the dependent variable to examine whether cognitive reserve was associated with stimulus evaluation speed and whether this association was moderated by working memory. In contrast to the amplitude findings, CR total score was not robustly associated with P300 latency in any cognitive status group, and none of the theoretically relevant CR or CR × WM latency effects survived FDR correction.

In HC, neither CR nor CR × WM interaction showed reliable associations with P300 latency. The CR effect was small, and its confidence interval crossed zero (*B* = 9.96, 95% CI [−55.65, 80.66]). The CR × WM interaction was also not supported (*B* = −20.65, 95% CI [−101.71, 37.41]). Similarly, in MCI, the CR effect was not robust (*B* = 24.90, 95% CI [−3.97, 74.86]) and the CR × WM interaction was not reliable (*B* = −16.60, 95% CI [−84.51, 29.53]). In MoCI, estimates were larger but highly uncertain, with wide confidence intervals crossing zero for both CR (*B* = −62.08, 95% CI [−317.63, 62.86]) and the CR × WM interaction (*B* = −5.76, 95% CI [−150.25, 88.97]). Thus, primary latency analyses using CR total did not provide robust evidence that cognitive reserve or WM-dependent reserve expression was associated with P300 latency.

Because CR total score was not associated with P300 latency in any group, additional exploratory models were conducted in the MoCI group to examine whether specific cognitive reserve domains related to processing speed. Given that P300 latency reflects stimulus evaluation speed rather than neural resource allocation, exploratory analyses focused on CRIq domains most directly related to sustained cognitive demands, namely occupational activity and education.

CR occupation was not reliably associated with P300 latency in HC (*B* = −11.25, 95% CI [−75.41, 53.94]) or MCI (*B* = 19.75, 95% CI [−16.82, 65.24]). In MoCI, higher CR occupation showed a negative coefficient in the final model (*B* = −93.03) and explained 21% unique variance in P300 latency. However, the confidence interval was wide and crossed zero (95% CI [−340.02, 27.40]), and the effect did not survive FDR correction (*p*FDR = 0.284). The CR occupation × WM interaction was not reliable in any group. Thus, although occupational reserve showed a comparatively larger exploratory association with P300 latency in MoCI, this effect was not robust under the bootstrapped and FDR-corrected inferential framework. CR education showed a similar but non-robust pattern in MoCI (*B* = −121.93, 95% CI [−379.51, 121.17]), while the corresponding MCI effect was also not reliable (*B* = 21.39, 95% CI [−18.17, 60.34]). The CR education × WM interaction was not reliable in the education models. Overall, P300 latency effects were inconsistent and less stable than amplitude effects, suggesting that latency was a less sensitive electrophysiological marker of cognitive reserve in the present sample. Because the primary Pz latency models did not provide robust evidence for CR-related or WM-dependent effects, and because latency was treated as a secondary electrophysiological outcome, no further adjacent-electrode or whole-sample interaction analyses were conducted for latency in order to limit additional exploratory testing.

## 4 Discussion

The present study investigated associations between cognitive reserve, working memory capacity and P300 electrophysiological measures in older adults across cognitive status groups. The findings suggest cognitive-status-related differences in how cognitive reserve associates with attention-related neural processing. In healthy older adults, higher cognitive reserve was associated with larger P300 amplitude, a pattern that may be consistent with more effective engagement of task-related neural resources, although this effect should be interpreted cautiously because it did not survive multiple test correction. In individuals with MCI, the association between cognitive reserve and P300 amplitude appeared to vary as a function of working memory capacity, suggesting possible WM-dependent modulation of reserve-related processing. In the moderate cognitive impairment cognitive reserve and the CR-WM interaction were not reliably associated with P300 amplitude, and estimates were highly uncertain. Overall, these findings provide preliminary evidence that CR-related electrophysiological associations may differ across cognitive status groups, while underscoring the need for cautious interpretation, particularly for the small MoCI group.

The finding that higher cognitive reserve was associated with larger P300 amplitude in healthy older adults is consistent with the interpretation of P300 amplitude as an index of neural efficiency in aging (Polich, 2007; Demirayak et al., 2023; Mendes et al., 2022) and with the view that P300 amplitude reflects the engagement of task-related attentional resources during stimulus evaluation and context updating (Polich, 2007; Gu et al., 2017, 2018). Within the cognitive reserve framework, this pattern may indicate that individuals with higher reserve showed more effective engagement of attention-related neural processing during task completion (Stern et al., 2019, 2023). Because oddball task accuracy and reaction time did not differ significantly across groups, the amplitude association is unlikely to reflect gross differences in overt task performance. However, given that P300 amplitude may also vary with attentional allocation, task engagement, and cognitive effort, this finding should be interpreted as consistent with, rather than definitive evidence of, reserve-related neural efficiency.

Importantly, the CR-P300 association in healthy older adults was not moderated by working memory capacity. This pattern suggests that in cognitively healthy aging, reserve-related differences in task-related neural processing are less dependent on the availability of additional working memory resources. This interpretation is broadly consistent with cognitive reserve accounts proposing that, when cognitive functioning is relatively preserved, reserve may be expressed through more effective or economical task-related processing rather than through recruitment of additional cognitive resources (Stern et al., 2019). Related functional neuroimaging studies have shown that individuals with higher reserve may exhibit reduced task-related activation, which has been interpreted as more economical neural processing (Steffener and Stern, 2012). The present findings extend this literature by suggesting that P300 amplitude may provide a temporally precise electrophysiological marker of reserve-related variation in attention-related processing during healthy aging.

In the MCI group, the pattern differed from that observed in healthy older adults. Higher cognitive reserve was not positively associated with larger P300 amplitude at mean working memory capacity. Instead, the CR-P300 association appeared to vary as a function of the available working memory resources. This pattern suggests that, in MCI, reserve-related differences in attention-related neural processing may be more closely linked to the availability of preserved working memory resources. However, because the group-stratified MCI effects did not survive multiple test correction, this finding should be interpreted cautiously as possible WM-dependent modulation of reserve-related processing rather than as direct evidence of cognitive reserve compensatory mechanism.

This conditional pattern is compatible with cognitive reserve models proposing that, when neural or cognitive integrity is reduced, reserve-related benefits may depend more strongly on the availability of preserved cognitive resources (Stern et al., 2019; Cabeza et al., 2018). In the present study, model-predicted values suggested that individuals with MCI and higher working memory capacity showed a more positive association between cognitive reserve and P300 amplitude, whereas this association was weaker or negative among those with lower working memory capacity. Importantly, the whole-sample group interaction model provided corrected evidence that the CR-P300 amplitude association differed between HC and MCI, supporting the view that reserve-related electrophysiological associations were not uniform across cognitive status groups. This HC-MCI contrast suggests that, relative to healthy aging, reserve-related neural processing in MCI may be less directly expressed through overall P300 amplitude and more contingent on the availability of preserved working memory resources.

These findings are consistent with electrophysiological evidence showing altered P300 dynamics in MCI and other prodromal stages of cognitive decline, where amplitude changes may reflect altered attentional resource allocation arising from reduced neural integrity, compensatory engagement, or both (Cespón et al., 2018; Khachatryan et al., 2021). Importantly, working memory was assessed using an independent visuospatial task rather than derived from the oddball task itself. This supports the interpretation that working memory acted as a moderating cognitive resource and not simply reflecting variation in task difficulty.

In individuals with moderate cognitive impairment, neither cognitive reserve nor its interaction with working memory was reliably associated with P300 amplitude. The estimates were imprecise indicating substantial uncertainty in this group. From a theoretical perspective, the absence of detectable CR-related amplitude associations may be compatible with accounts suggesting that reserve-related compensatory processes become less effective when cognitive and neural integrity are more substantially compromised (Stern, 2012; Cabeza et al., 2018). Within this framework, cognitive reserve may delay the clinical manifestation of cognitive decline but may not indefinitely support efficient or compensatory task-related neural processing. However, given the small MoCI sample, this pattern should be interpreted as exploratory.

A central contribution of this study lies in distinguishing P300 amplitude and latency as functionally different electrophysiological indices. Whereas P300 amplitude showed clearer associations with cognitive reserve and working memory, latency effects were inconsistent and generally non-robust. This distinction is consistent with ERP literature suggesting that P300 latency is more closely related to stimulus evaluation timing, whereas amplitude reflects the allocation of attentional and cognitive resources during task-relevant processing (Polich, 2007; Mendes et al., 2022; Kao et al., 2022). In the present study, this pattern suggests that CR-related variation was more strongly expressed through amplitude-based attentional resource allocation than through latency-based differences in stimulus evaluation timing.

Exploratory domain-level analyses in moderate cognitive impairment suggested that occupational reserve may be associated with P300 latency. However, these effects were not supported by bootstrapped confidence intervals and should therefore be interpreted cautiously. Rather than indicating a robust reserve-related latency effect, this pattern may point to greater variability in stimulus evaluation timing among individuals with more pronounced cognitive impairment. Overall, latency findings were less consistent than amplitude findings and did not provide stable evidence for CR-related differences in processing speed or temporal efficiency.

Methodologically, the present findings highlight the importance of integrating multidimensional reserve measures, independent cognitive resource assessments, and temporally precise neural markers within the same individuals. Many previous studies have relied on single reserve proxies, most commonly education, which may obscure the multidimensional nature of reserve (Stern, 2009; Khachatryan et al., 2021). By combining CRIq indices, working memory performance, and ERP measures, the present study provides a more nuanced account of how cognitive reserve relates to task-related neural processing during aging and neurocognitive impairment.

Theoretically, the present findings are compatible with the view that cognitive reserve may be expressed differently across cognitive status groups, with P300 amplitude appearing more sensitive than latency to reserve-related variation in task-related attentional processing. The results suggest that CR-related electrophysiological associations may vary depending on cognitive status and the availability of working memory resources. Electrophysiological measures sensitive to attentional resource allocation, such as P300 amplitude, may therefore be useful for examining how cognitive reserve relates to task-specific neural processing in aging and neurocognitive impairment.

Several limitations should be acknowledged. First, the relatively small sample size in the moderate cognitive impairment group limits statistical power, model stability and the precision of parameter estimates, particularly for regression models including interaction terms. Although bootstrapped confidence intervals were used to reduce reliance on parametric assumptions, bootstrapping does not overcome the limitations of small group sample. Therefore, replication in larger clinical samples is needed. Second, the cross-sectional design precludes direct inferences about longitudinal changes in reserve expression. Although the findings suggest that CR-related electrophysiological associations may differ across cognitive status groups, longitudinal studies are essential to determine whether these patterns reflect within-individual transitions in reserve-related processing over the course of cognitive decline.

Third, the primary ERP analyses focused on P300 amplitude and latency at the Pz electrode. This choice was consistent with the expected parietal distribution of the P3b component and reduced the number of primary statistical tests. However, it limits conclusions about broader spatial patterns of reserve-related neural activity. Topographical maps and adjacent-electrode sensitivity analyses at P3 and P4 provided additional spatial context, but future studies with larger samples should examine whether similar CR-related effects are observed using ROI-based and whole-scalp approaches. In addition, P300 amplitude was quantified using peak amplitude, which is commonly used in ERP research (Luck, 2014) but may be more sensitive to noise than mean-amplitude measures, particularly in modest samples. Future studies should examine whether the present findings are replicated using alternative amplitude quantification methods, including mean amplitude. Also, incorporating additional cognitive domains beyond working memory may further elucidate which resources most strongly support reserve-related processes at different levels of decline.

Fourth, because the P300 was elicited using an auditory oddball task, the findings should be interpreted as reflecting the relationship between cognitive reserve and attention-related neural processing during this specific task context, rather than the neural expression of cognitive reserve more generally. Formal audiometric thresholds were not obtained, and although participants were screened for significant sensory difficulties and were required to perceive the auditory stimuli, unmeasured individual differences in hearing acuity may have influenced auditory ERP responses.

Finally, the cognitive reserve assessment was administered after the main cognitive and EEG assessments, either in person as the final assessment of the study visit or during a separate structured telephone interview when participants reported fatigue or preferred later completion. Although this approach may have reduced participant burden and fatigue during the main testing session, the use of different administration routes may have introduced some variability in assessment context. However, the same structured CRIq administration and scoring procedures were used in both formats. Future studies may benefit from using a fully standardized administration format and from combining questionnaire-based reserve indices with additional objective indicators of lifelong cognitive, occupational, and social engagement.

## 5 Conclusion

In summary, the present study suggests that cognitive reserve is differentially associated with P300 amplitude across cognitive status groups. In healthy aging, higher cognitive reserve was associated with larger P300 amplitude, consistent with more effective engagement of task-related attentional resources. In individuals with mild cognitive impairment, reserve-related P300 amplitude patterns appeared more dependent on working memory capacity, suggesting that preserved cognitive resources may shape how reserve is expressed in attention-related neural processing. In moderate cognitive impairment, CR-related amplitude associations were not reliably detected, although this subgroup should be interpreted cautiously given the small sample size and imprecise estimates. Overall, these findings support the utility of P300 amplitude as a task-specific electrophysiological marker for examining links between cognitive reserve, working memory, and attention-related neural processing in aging and neurocognitive impairment.

## Data Availability

The raw data supporting the conclusions of this article will be made available by the authors, without undue reservation.
